# From ambivalence to agency: parents’ conceptions of a theory-based behavioural intervention to prevent dental caries in their preschool children – a qualitative study

**DOI:** 10.1186/s12903-026-07864-z

**Published:** 2026-02-11

**Authors:** Sara Björns, Marlene Makenzius, Peter Lingström, Eva-Karin Bergström

**Affiliations:** 1 Department of Cariology, Institute of Odontology, Sahlgrenska Academy, University of Gothenburg, Box 450, Gothenburg, SE-405 30 Sweden; 2 Department of Preventive and Community Dentistry, Public Dental Service, Gothenburg, Region Västra Götaland Sweden; 3 Department of Health Sciences, Mid Sweden University, Östersund, Sweden; 4 Department of Global Public Health, Karolinska Institutet, Stockholm, Sweden; 5 Department of Women’s and Children’s Health, Karolinska Institutet, Stockholm, Sweden

**Keywords:** Dental caries, Empowerment, Family health, Health promotion, Public health dentistry, Self-efficacy, Qualitative research

## Abstract

**Background:**

Dental caries remains one of the most prevalent chronic conditions in preschool children and disproportionately affects families in socioeconomically vulnerable contexts, despite publicly funded preventive dental care. In Region Västra Götaland, Sweden, a theory-based behavioural intervention delivered by health promoters was introduced to support families of children at elevated caries risk. The aim of this study was to describe the different ways in which parents conceive a health-promoter-led, theory-based behavioural intervention to prevent dental caries in their preschool-aged children.

**Methods:**

A qualitative study using individual, semi-structured interviews were conducted in ten public dental clinics in Region Västra Götaland, Sweden (March 2023–July 2024). Interviews were analysed using phenomenographic analysis to capture variation in parental conceptions. Ten parents (eight women, two men) of three-to-six-year-old children at elevated caries risk completed two or more counselling sessions with university-trained health promoters. Interviews (30–60 min) were audio-recorded, transcribed verbatim and analysed inductively to capture variation in parental perceptions. Flexible delivery (digital/in-clinic) and interpreter support were made available.

**Results:**

Three themes were identified: (1) *an invitation met by ambivalence and fear of judgement*, (2) *empowered alliance through personalised support* and (3) *active choices through parental agency*. These themes coalesced into the overarching theme ‘from ambivalence to agency: embracing health-promoting behaviour’.

**Conclusions:**

Parents conceived the health-promoter-led, theory-based behavioural intervention as non-judgemental, culturally responsive and practically useful. By fostering relational safety and providing actionable tools, the intervention appeared to strengthen parental self-efficacy and catalyse family-level behavioural change. This shift, while modest in scale, may represent a necessary step towards equitable and sustainable oral-health promotion.

## Background

Health is based on an interplay of structural and individual-level determinants, including socioeconomic status, healthcare provision and daily behaviours [1]. Dental caries is associated with these determinants and remains the most common non-communicable disease among children worldwide, including in Sweden [2, 3]. In Sweden, dental care for children is publicly funded and organised by the country’s 21 regions, which are responsible for providing preventive, restorative and emergency services, financed through taxes. Despite a strong focus on prevention, social inequalities in oral health persist, particularly among children from disadvantaged backgrounds [4, 5]. Nordic register-based studies show that caries remains socially patterned across adolescence despite universal child dental care, and that caries also clusters strongly within families. Together, these findings support prevention strategies that move beyond the individual child and explicitly target family and socioeconomic contexts [4–7]. Approximately 83% of 6-year-olds in Västra Götaland are caries-free. However, in certain geographic areas, this proportion varies from 60% to 95% and remains stable year after year, reflecting persistent and localised disparities in oral-health outcomes related to socioeconomic factors [6].

Oral health promotion for families with young children can be understood as a relational process shaped by communication, trust, and access to supportive care contexts. Swedish qualitative research shows that migrant parents’ oral health literacy is influenced by how oral health information is accessed, interpreted, and assessed for credibility, highlighting the importance of culturally appropriate dialogue in preventive dental care [8]. Studies from family centre settings further indicate that meeting dental professionals in non-clinical, interprofessional environments is perceived as enabling more equal dialogue, trust, and parental learning [9]. Together, these studies indicate that preventive oral health interventions may benefit from being embedded in accessible settings that support trust, cultural responsiveness, and parental agency.

In Region Västra Götaland, all children are offered a routine dental check-up as part of a structured risk-assessment protocol that categorises them according to their likelihood of developing caries. Those identified as being at elevated risk are subsequently offered participation in the Recommended Programme for Caries Treatment (RPCT), which is delivered by dental hygienists and dental assistants through preventive measures. The RPCT focuses on early detection, individualised follow-ups and tailored guidance aimed at reducing the need for invasive treatment. Such treatment not only imposes financial burdens on the healthcare system but also adversely affects children’s quality of life, academic performance and future oral-health trajectories [5, 7]. Given the preventable nature of dental caries, early childhood is a key developmental period for establishing enduring oral-health behaviours that can mitigate future disease burden [5, 6].

In response to persistent oral-health inequalities, Region Västra Götaland initiated a three-year regionally commissioned project in 2021 that introduced a new occupational role known as ‘health promoter’ within the public dental-care system. Internationally, comparable roles are often described as ‘community health workers’, who typically have varied educational backgrounds and practice-oriented training; in contrast, the health promoters in this study held university degrees in behavioural science or public health and delivered theory-based behavioural counselling embedded within the public dental-care system [10, 11]. As part of the health promoter initiative, a targeted behavioural intervention was implemented at eleven clinics selected for their consistently high prevalence of dental caries among 6-year-old children. Unlike traditional dental professionals, health promoters have no formal education in odontology. Instead, they hold academic degrees in areas such as behavioural science, health promotion or public health, which provide them with competencies in theory-based methods designed to support sustained changes in health behaviours. This approach is consistent with national guidelines that emphasise the importance of theory-driven interventions in preventive and promotive care [12]. The health promoters engaged with families of children aged three–six years who were classified as having elevated caries risk, delivering a structured theory-based behavioural intervention. The health promoters received specialised training in oral-health promotion and collaborated with dental professionals closely working alongside their clinical expertise, thereby supporting an integrated model of care. The intervention was conceptually grounded in social cognitive theory [13] and informed by the principles of motivational interviewing [14], both of which underscore the importance of self-efficacy, behavioural reinforcement and social modelling in influencing health-related behaviours. Employing an empowerment-based approach, the health promoters aimed to reshape parental attitudes and facilitate behavioural change within the family context. Given the significant role of caregiver practices in shaping children’s oral health, family-oriented interventions are recognised as essential [5, 7, 8, 15].

A health economic evaluation demonstrated that a theory-based behavioural intervention, delivered by health promoters, showed a statistically significant reduction in caries prevalence and increased cost-effectiveness compared with the RPCT over a three-year timeframe [16]. Yet the intervention’s acceptability, perceived usefulness and impact on family dynamics remains unexplored. The success and sustainability of such interventions depend on their resonance with the perceptions and lived experiences of the families they serve. Understanding how parents perceive these theory-based consultations is therefore essential for assessing whether the intervention meets familial needs, facilitates sustainable behavioural change and mitigates barriers to achieving good oral health for the families and their young children. Therefore, the aim of this study was to describe the different ways in which parents conceive a health-promoter-led, theory-based behavioural intervention to prevent dental caries in their preschool-aged children.

## Methods

### Study design

A qualitative study design was selected to explore the variation in parental perceptions of a theory-based behavioural intervention delivered by health promoters. The study adhered to the Consolidated Criteria for Reporting Qualitative Research (COREQ) guidelines [17]. Although grounded in a common risk factor perspective on health, the intervention was operationalised through a risk profile based on assessed risk for oral disease. A purposive sample of ten parents (eight women and two men) were recruited, all of whom had children aged three to six years that had been identified during routine dental examinations as being at increased risk of caries. The inclusion criteria required the parents to have participated in a minimum of two consultations with a health promoter. Participants represented linguistic and cultural diversity; most spoke Swedish as a second language, and one interview was conducted with interpreter support. The linguistic diversity of participants reflects the catchment areas of the selected clinics, which serve populations with higher proportions of families with migration backgrounds. Eligible families were identified by the health promoters during the intervention period and were selected based on their perceived ability to contribute diverse perspectives to this study. To ensure representation across all ten participating clinics, each of the five health promoters was instructed to invite two parents from the health promoter’s assigned clinic. Two respondents spoke Swedish as their native language, seven participants spoke Swedish as a second language and one interview was facilitated by an interpreter.

### Setting

The interviews were conducted between March 2023 and July 2024 and occurred in Region Västra Götaland, Sweden. Participants were offered a choice between digital or in-person individual interviews; all selected digital formats (video or telephone). Digital interviews were conducted via Teams or FaceTime allowing real-time interaction including both audio and video. Interviews were conducted by the author (SB), a dental public health researcher unaffiliated with the participants’ clinical care. Participants were informed of their right to request an interpreter for free, and one interview was conducted with professional interpreting support to ensure communication in the participant’s preferred language. Interviews lasted 30–60 min; they were audio-recorded after written informed consent was obtained and then transcribed verbatim by SB. A semi-structured interview guide developed for this study (Supplementary) that began with an explanation of the study purpose. This was followed by open-ended questions exploring participants’ perceptions of the intervention’s purpose, delivery, relational dynamics and perceived impact on family health behaviours.

### Ethical considerations

Ethical approval was granted by the Swedish Ethical Review Authority (No. 2022-06428-01). Ethical considerations were made during the planning and implementation of the study, including requirements concerning information, consent, use and confidentiality. Participants received both oral and written information about the study and its purpose prior to the start of the interview. They were informed that participation was voluntary and that they could decline, withdraw from the study at any time or choose not to answer specific questions without providing a reason.

### Data analysis

**Fig. 1:**
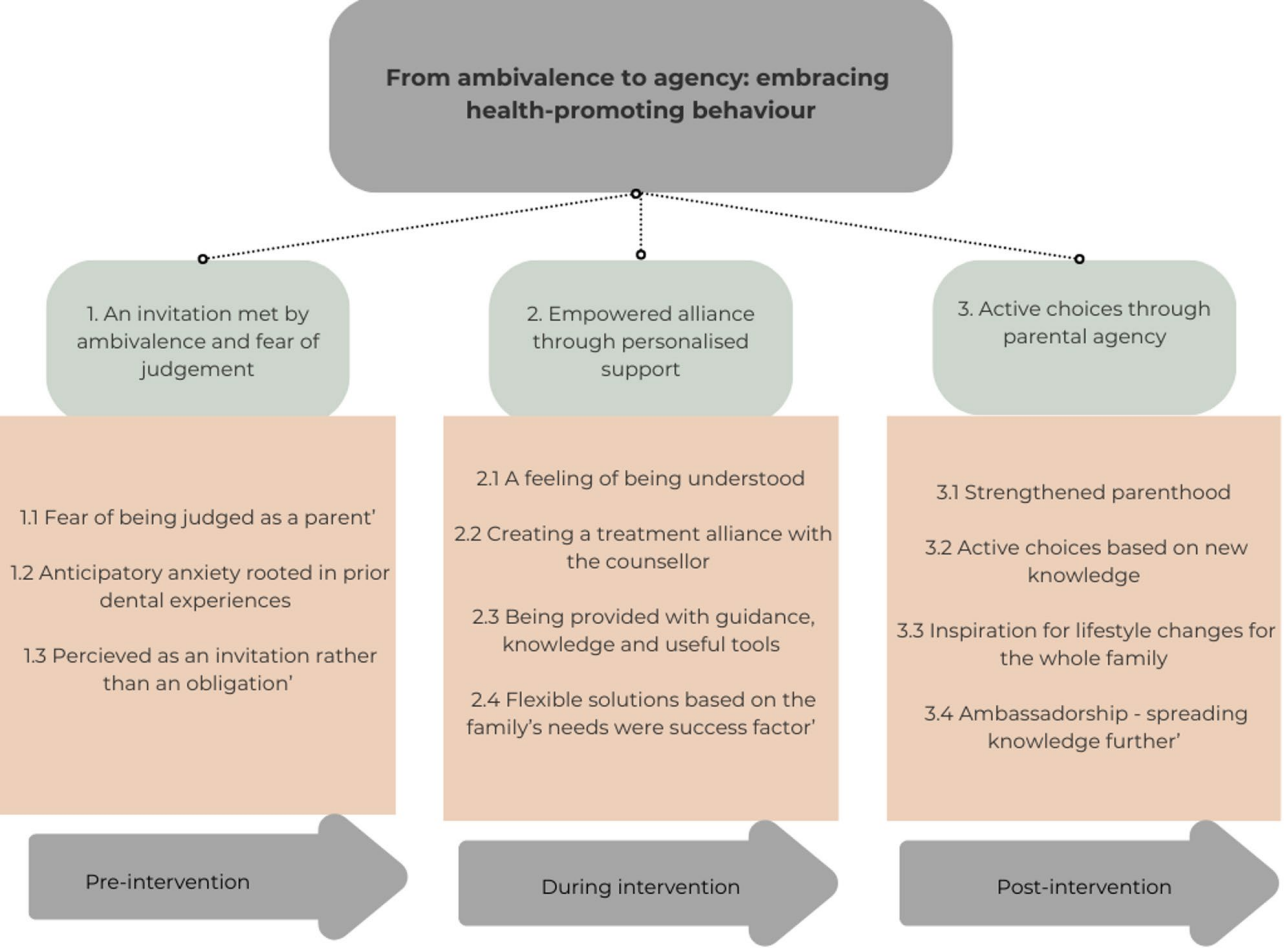
Themes and categories illustrating variation in parents’ conceptions of a health-promoter-led, theory-based behavioural intervention to reduce caries risk among children aged three to six years, structured across pre-intervention, during-intervention and post-intervention phases

The analysis took an inductive phenomenographic approach [18, 19], based on Alexandersson’s [20] four steps. First, the transcripts were read thoroughly several times to obtain an overall impression of the material. Next, similarities and differences in the material were noted. In the third step, statements were sorted into descriptive categories of conceptions. In the final step, the categories were reflected upon, and three themes emerged; these then resulted in an overarching theme articulating how parents experienced the theory-based behavioural intervention with health promoters in the Public Dental Service (Fig. 1). To enhance credibility, SB and EKB independently coded a purposive subset of transcripts, prioritising interviews where Swedish was a second language, and the meaning was more analytically challenging. Coding differences were discussed until consensus was reached and used to refine the developing categories and themes. Illustrative quotes are provided alongside each thematic category to increase transparency and support the analytical findings.

## Results

The parents’ conceptions of the theory-based behavioural intervention to prevent dental caries were framed into three descriptive themes: (1) ‘an invitation met by ambivalence and fear of judgement’, (2) ‘empowered alliance through personalised support’ and (3) ‘active choices through parental agency’. Each theme comprises several categories that reflect variations in how parents understood and experienced the intervention. The three themes were synthesised into an overarching theme: ‘from ambivalence to agency: embracing health-promoting behaviour’ (Fig. 1).

### Theme 1: *an invitation met by ambivalence and fear of judgement*

The first theme, *an invitation met by ambivalence and fear of judgement*, captures parents’ initial conceptions of the intervention prior to meeting with the health promoters. These conceptions were characterised by ambivalence and fear of judgement as related but distinct experiences. Ambivalence reflected simultaneous openness and hesitation towards participation, while fear of judgement was linked to concerns about being evaluated as parents, often informed by prior encounters with dental care. The theme consists of three categories: ‘fear of being judged as a parent’, ‘anticipatory anxiety rooted in prior dental experiences’ and ‘conceived as an invitation rather than an obligation’.

#### *Category 1.1: ‘fear of being judged as a parent’*

Parents entered the consultation with a pronounced apprehension about being judged for their caregiving practices. One parent explained,

So, I was a little nervous, I felt, but it felt good at the same time; I felt like, ‘Oh, now she’s probably going to judge and criticise me’, but she didn’t. She was absolutely wonderful … (Respondent 10).

This statement illustrates the parents’ initial fear of criticism, which was subsequently alleviated by the health promoter’s empathetic approach.

#### *Category 1.2: ‘anticipatory anxiety rooted in prior dental experiences’*

A second category reflected parents’ concern regarding the state of their children’s oral health and their previous experiences with dentists. One parent noted,

“The most important thing when we go to the health centre is that he doesn’t cry and starts to feel safe. That he starts to take fluoride. It makes a big difference for me and his dad. Before [when we had a dentist appointment], we would start thinking and worrying for ten days, thinking, ‘What can we do? What should we do about that place?’ [The intervention] makes a big difference. It has changed his situation so much.“ (Respondent 6).

#### Category 1.3: ‘conceived as an invitation rather than an obligation’

The third category concerns how parents conceived the intervention as an invitation rather than an obligation. One parent stated,

“Yes. It happened in such a way that my son had caries or had cavities on some teeth. And it was more like an offer that I received … “( Respondent 3).

Here, the parent explained that the intervention was an offer received after a routine examination where multiple dental caries were detected.

### Theme 2: *empowered alliance through personalised support*

The second theme, *empowered alliance through personalised support*, includes the parents’ conceptions of the theory-based behavioural intervention itself. This theme consists of four categories: ‘a feeling of being understood’, ‘creating a treatment alliance with the counsellor’, ‘being provided with guidance, knowledge and useful tools’ and ‘flexible solutions based on the family’s needs were a success factor’.

#### *Category 2.1: ‘a feeling of being understood’*

During the consultation, many parents reported a sense of being understood by the health promoter. One parent remarked,

“But yeah, but that was the thing about it, that I didn’t feel like she was judging me, but she was more understanding, and that’s what I actually liked about her.” (Respondent 10).

Here, the parent highlights the significance of an empathetic, non-judgmental dialogue.

#### *Category 2.2: ‘creating a treatment alliance with the counsellor’*

Parents also emphasised the establishment of a collaborative relationship with the health promoter. As one parent explained,

“It was great, and it felt like there was, there was someone who was on my side and kind of supporting me.” (Respondent 5).

This parent felt a sense of alliance that contributed to a supportive consultation environment, facilitating open communication.

#### *Category 2.3: ‘being provided with guidance, knowledge and useful tools’*

The consultation provided concrete guidance and practical tools, enabling parents to make informed decisions regarding their children’s oral health. One participant observed,

“The second tip was about bread that contains sugar, and she told me about the Keyhole [*nyckelhålet*] label on products [a Nordic nutrition label marking healthier products]. I started buying products with a Keyhole label.” (Respondent 5).

This category reflects how the participants described the practical guidance and tools offered during the consultation as helpful in supporting decisions related to their children’s oral health.

#### *Category 2.4: ‘flexible solutions based on the family’s needs were success factor’*

This category shows how participants described the flexibility of the consultation format as a positive aspect of the intervention that enabled adaptation to the needs and circumstances of individual families. One parent commented,

“[During the] digital meeting, it was exactly as if I were there [in person] … It was the same quality … I don’t think it was strange, it was great, I think so.” (Respondent 2).

This parent’s comment emphasises that the adaptability of the intervention (i.e. whether it was delivered in person or digitally) was experienced as a strength.

### Theme 3: *active choices through parental agency*

The third theme, *active choices through parental agency* contains parents’ perceptions after the theory-based behavioural intervention with a health promoter. This theme consists of four categories: ‘strengthened parenthood’,‘active choices based on new knowledge’, ‘inspiration for lifestyle changes for the whole family’ and ‘ambassadorship - spreading knowledge further’.

#### *Category 3.1: ‘strengthened parenthood’*

Following the consultation, parents reported an enhanced sense of self-efficacy and empowerment in their caregiving roles. One parent remarked,

“Now we stop it; even if she cries in the store, we don’t listen. At first, that was our response. But she also started to accept that it was every other Friday that she gets candy. Even if we go to the store now, there’s less screaming. Now she doesn’t act like before, when she screamed that she had to have candy. Even when she says no, if she cries all day, she gets it every other Friday.” (Respondent 9).

In this quotation, the parents explain how the intervention helped reinforce their parental role, fostering both personal and familial well-being.

#### *Category 3.2: ‘active choices based on new knowledge’*

Acquiring new, practical insights prompted the parents to adopt more proactive measures in their daily routines. One parent stated,

“… And so we talked about such a multivitamin without sugar that would be best for my child, and something else. What was it? A jam. Because my child craves jam a lot.” (Respondent 2).

The parents described how the actionable recommendations they were given encouraged them to make deliberate and healthier choices.

#### *Category 3.3: ‘inspiration for lifestyle changes for the whole family’*

The parents described how the consultations acted as a catalyst for broader changes in habits that extended beyond the immediate focus on oral health and affected the whole family. One participant noted,

“The meeting was just about my daughter, but it affected the whole family.” (Respondent 9).

As in this example, the knowledge the parents gained led them to re-evaluate their family’s daily habits.

#### *Category 3.4: ‘ambassadorship - spreading knowledge further’*

The parents described how they took on an ambassador role, enthusiastically sharing the new insights and practical tips they had gained with their friends, relatives and community:

“I’ve told many of my friends [about the things] I didn’t know – like, I’ve been brushing my teeth, little things like that, wrong my whole life. And I was lucky that I haven’t lost many teeth.” (Respondent 2).

## Discussion

The parents’ conceptions of the health-promoter-led, theory-based behavioural intervention to prevent dental caries were framed into three descriptive themes: (1) ‘an invitation met by ambivalence and fear of judgement’, (2) ‘empowered alliance through personalised support’ and (3) ‘active choices through parental agency’. Taken together, the findings suggest that a health-promoter-led approach, grounded in social cognitive theory and motivational interviewing, was able to foster a non-judgmental atmosphere and empower families with targeted practical guidance. This guidance was typically operationalised as specific, feasible micro-changes in daily routines, such as identifying lower-sugar food options, adjusting snack frequency, and selecting sugar-free alternatives. This practical guidance was described by the parents as strengthening the capacity beyond oral hygiene by supporting limit setting, comfort and caregiving routines, and helping parents navigate everyday situations where food and sweets functioned as consolation, reward or relational care. In this way, dietary advice was framed within the family’s meaning making and parenting practices, rather than as isolated nutrition rules.

The fear of judgment and uncertainty reported by the parents prior to their visit is consistent with prior research showing that stigma or apprehension about parental practices can inhibit health-seeking behaviours [21]. However, the encounters with the health promoters were perceived as non-judgemental, personalised and flexible – qualities associated with successful motivational interviewing [14]. The parents’ accounts described a transition from ambivalence to agency, with the intervention facilitating deliberate health-related decisions and broader lifestyle shifts, including practices such as limiting sugary snacks and adopting more nutrient-dense, low-sugar dietary choices. These changes indicate a generalisation of behaviour beyond oral hygiene, an outcome congruent with social cognitive theory’s emphasis on reinforcement and modelling in diverse contexts [13]. Perceptions such as ‘feeling understood’ and ‘acting as ambassadors’ reflect not only increased self-efficacy but also the emergence of a relational context that enables the internalisation of new caregiving norms. This result is consistent with the findings of Alvenfors et al. [22], which emphasise the importance of alliance-building between dental professionals and caregivers as a prerequisite for translating knowledge into sustained behavioural change.

The health promoter role may be central to understanding the transition from ambivalence to agency observed in this study. Unlike traditional dental professionals, health promoters in this programme were university trained in behavioural science or public health and delivered structured counselling focused on behaviour change rather than clinical procedures. Although embedded within the public dental-care system, they did not occupy the same professional authority as dentists or dental hygienists, which may have reshaped the interactional dynamics of the consultation.

Consultations were typically conducted in neutral, non-clinical environments rather than treatment rooms, removing procedural cues associated with dental examination or intervention. This setting, together with the health promoters’ non-judgemental stance and personalised support, appeared to shift the focus of the dialogue away from the tooth as an isolated object of care towards broader aspects of everyday family life. Parents described discussing issues such as routines, limit-setting, rewards, and caregiving dynamics, enabling reflection on how oral-health practices are embedded within daily living.

Parents’ accounts of feeling supported by “someone on my side” suggest that the health promoters facilitated a relationally safe encounter characterised by practical problem-solving, open communication, and culturally responsive dialogue. This relational and contextual approach may help explain the reported increase in parental confidence in everyday limit-setting and the translation of oral-health guidance into broader, family-level routines. Theoretical frameworks such as relational autonomy can help interpret these findings. This concept suggests that individual agency is co-constructed through supportive, trust-based social relationships [20]. In this study, the non-judgemental stance of the health promoters appeared to create a ‘safe relational space’ in which parents could reflect on and recalibrate their caregiving practices in achievable ways while feeling respected. This finding complements social cognitive theory’s focus on self-efficacy by highlighting the social conditions that enable individuals to act on health-related intentions [13].

An intersectional perspective [24] can further inform our understanding of the findings. Several parents described navigating language barriers and cultural differences when accessing public services. Intersectionality theory helps explain why a culturally tailored intervention may better support engagement among diverse families, how generic caries prevention often under-serves marginalised groups, and why culturally responsive dialogue proved critical in the present case [25, 26]. Furthermore, the parents’ positive reception to practical guidance and flexible solutions resonates with literature emphasising the necessity of culturally and contextually adapted interventions for families from diverse backgrounds [7, 23, 24]. This study’s findings are also compatible with the salutogenic model: parents described how the intervention helped make oral-health tasks more comprehensible, manageable and meaningful, dimensions central to a sense of coherence [24].

At the micro level, the theme *active choices through parental agency* showed how parents reported tangible shifts such as adopting “Keyhole-labelled foods” and enforcing ‘candy only on Saturdays’, signalling a recalibration of day-to-day routines. These examples illustrate the enactment of the overarching theme, ‘from ambivalence to agency’, as families translated abstract guidance into tangible routines. These changes were enabled by meso-level dynamics, as captured in the theme *empowered alliance through personalised support*, where trust-based rapport with culturally responsive health promoters and the option of digital visits lowered logistical and communicative barriers. However, some accounts covered in the theme ‘an invitation met by ambivalence and fear of judgement’, reflected enduring concerns related to past healthcare experiences, which may continue to shape how preventive and promotive efforts are received and acted upon. Importantly, the interpreter-facilitated session was described as effective, suggesting that a relational alliance can be achieved across linguistic boundaries when delivery is culturally sensitive [27]. Thus, the findings from this study suggest that an integrated approach that reinforces micro-level behavioural shifts, optimises meso-level service delivery and addresses macro-structural inequities may lead to durable reductions in childhood caries risk.

Dental caries shares dietary, socioeconomic and behavioural determinants with obesity, type-2 diabetes and cardiovascular diseases. The observed reductions in sugary-snack frequency suggest potential collateral benefits across multiple non-communicable diseases consistent with the common risk-factor approach [28, 29]. Recent longitudinal evidence further supports this perspective, demonstrating that poor childhood oral health is associated with an increased incidence of atherosclerotic cardiovascular disease in adulthood, underscoring the long-term systemic relevance of early caries prevention [30]. Framing the intervention within a syndemic paradigm, where oral disease co-occurs and interacts biologically and socially with other conditions, may increase its relevance for inter-sectoral collaboration and resource allocation [23]. The ambassadorial role described by several parents may reflect peer-led social modelling, potentially amplifying the intervention’s reach beyond the immediate family and reinforcing social-cognitive-theory-based mechanisms of influence.

Overall, these findings suggest that culturally attuned, theory-based consultations can support parents in moving from externally motivated compliance to internally anchored agency. This shift, while modest in scale, may represent a necessary step towards equitable and sustainable oral-health promotion.

This study has limitations that should be noted. This phenomenographic enquiry was based on a region-specific sample of ten families, which was appropriate for depth but inevitably restricted the range of parental conception that could be captured. The sample was structured to ensure representation across all participating clinics, with approximately two parents recruited from each of the five clinics involved. Self-selection and awareness that the interviewer was linked to the dental service may have introduced social-desirability bias.

Interviews were conducted in Swedish, with professional interpreter support offered when needed; one interview was conducted with an interpreter in the participant’s preferred language. Most participants spoke Swedish as a second language. Linguistic diversity may have influenced how parents articulated emotions, meanings, and causal explanations, and some nuances may therefore have been attenuated during translation and transcription. All participants were families residing and actively engaged in everyday life in Sweden, and their accounts reflected lived experiences within the Swedish public service context. To strengthen analytic credibility and reduce the risk of misinterpretation, interviews in which Swedish was a second language were prioritised for independent double-coding. While minor lexical or grammatical irregularities occurred, the substantive meaning and contextual intent of participants’ accounts were consistently clear and readily interpretable.

Regarding implications and future research, the findings from this study have clear implications for both practice and policy. By demonstrating how culturally responsive, theory-based behavioural intervention can enhance parental agency and foster health-promoting routines beyond toothbrushing, this research supports the scale-up of health-promoter-led interventions within child dental-care services. The alignment with social cognitive theory and salutogenic principles suggests that behavioural counselling in dental contexts should not be narrowly confined to risk communication or compliance but instead framed within a broader relational and contextual understanding of families’ lived realities. In clinical practice, these insights can inform training modules that equip health promoters and dental teams to work relationally, motivationally and with attention to intersectional determinants of health.

Importantly, health promoters should be viewed as one component within an interprofessional team, where dental hygienists, dental assistants and dentists play a pivotal role in promotive and preventive care. Dental hygienists are uniquely positioned to integrate behavioural conversation into routine practice, bridging clinical prevention with person-centred communication. A collaborative approach in which health promoters, dental hygienists and other team members contribute complementary skills can maximise the impact of preventive and promotive strategies and ensure that interventions resonate with families’ daily realities.

At the policy level, the findings argue for stronger structural integration between oral-health services and broader child health or family support systems. This includes revisiting commissioning models to ensure that preventive and promotive services are not only reimbursed adequately but also designed in ways that allow for flexible, tailored and culturally sensitive encounters. Moreover, applying a syndemic perspective may enable oral-health interventions to be strategically embedded within cross-sectoral public-health strategies targeting diet, equity and early child development. Given the disproportionate burden of dental caries among socioeconomically and culturally marginalised groups [4–6], this approach could increase both efficiency and equity in resource allocation.

Further research is warranted to build on these findings. Interviews with clinical personnel involved in the delivery of such interventions could provide a more complete picture of implementation dynamics, role clarity and perceived enablers or barriers within everyday practice. A complementary retrospective epidemiological analysis of children’s dental records, comparing those exposed to the health-promoter intervention with controls, could help assess whether the reported behavioural shifts translate into measurable clinical outcomes over time. Such mixed-method triangulation would strengthen the evidence base and inform future guidelines for scalable, equity-oriented caries-prevention programmes.

## Conclusions

This study contributes new insights into how parents perceived the value and delivery of a theory-based behavioural intervention delivered by health promoters. This intervention was perceived as non-judgemental, flexible and practically useful, supporting parental self-efficacy and encouraging broader family-level change.

## Data Availability

De-identified interview transcripts are available from the corresponding author upon reasonable request.

## Abbreviations

COREQ: Consolidated Criteria for Reporting Qualitative Research
MI: Motivational interviewing
RPCT: Recommended Programme for Caries Treatment
SCT: Social cognitive theory
SKaPa: Swedish Quality Register for Caries and Periodontitis
